# Supplementary material for: Animal Virus Ecology and Evolution Are Shaped by the Virus Host-Body Infiltration and Colonization Pattern
Source: Pathogens. 2019 May 25;8(2):72. doi: 10.3390/pathogens8020072 (PMC6631033; doi:10.3390/pathogens8020072)
Supplement: Supplementary file 1 [file pathogens-08-00072-s001.zip › SUPPLEMENTARY MATERIALS Figure S1c.docx]

# Supplementary Materials

**Figure S1c: Literature sources Figure S1a.**

**(Accessed on 15 February 2019)**

1. World Organisation for Animal Health, “Chapter 2.5.10. Equine viral arteritis (Infection with Equine arteritis virus)” in *World Organisation for Animal Health (OIE) Manual of diagnostic tests and vaccines for terrestrial animals*. **2013**, pp. 1-17. Available at <http://www.oie.int/fileadmin/Home/eng/Health_standards/tahm/2.05.10_EVA.pdf>
2. Spickler, A.R.; **2009**. Equine Viral Arteritis. Retrieved from <http://www.cfsph.iastate.edu/DiseaseInfo/>
3. World Organisation for Animal Health, “Appendices IV and V, PRRS: the disease, its diagnosis, prevention and control.” in *Report of the OIE ad hoc group on porcine reproductive respiratory syndrome*, *Paris, 9-11 June 2008.***2008.**Retrieved from <http://www.oie.int/fileadmin/Home/eng/Our_scientific_expertise/docs/pdf/PRRS_guide_web_bulletin.pdf>
4. World Organisation for Animal Health. General Disease Information Sheets, Porcine reproductive and respiratory syndrome. **2011.** Retrieved from <http://www.oie.int/doc/ged/D13986.PDF>
5. van den Berg, T.P.; Eterradossi,N.; Toquin, D.; Meulemans, G. Infectious bursal disease (Gumboro disease). *Rev sci tech Off int Epiz*. **2000**, *19(2)*, 527-543. Retrieved from <https://www.oie.int/doc/ged/d9315.pdf>
6. Food and Agriculture Organization of the United Nations Secretariat of the Pacific Community, “B309 - Infectious bursal disease” in *AHP Manual – Reference Guide for Animal Health Staff,* Fediaevsky, A., Ed. **2002.** Retrieved from <http://lrd.spc.int/ext/Disease_Manual_Final/b309__infectious_bursal_disease.html>
7. Food and Agriculture Organization of the United Nations Secretariat of the Pacific Community, “B301 – Avian infectious bronchitis” in *AHP Manual – Reference Guide for Animal Health Staff,* Fediaevsky, A., Ed. **2002.** Retrieved from <http://lrd.spc.int/ext/Disease_Manual_Final/b301__avian_infectious_bronchitis.html>
8. World Organisation for Animal Health, “Chapter 2.3.2. Avian infectious bronchitis” in *World Organisation for Animal Health (OIE) Manual of diagnostic tests and vaccines for terrestrial animals*, OIE. **2018**, pp. 1-14. Retrieved from <http://www.oie.int/fileadmin/Home/eng/Health_standards/tahm/2.03.02_AIB.pdf>
9. Department of Veterinary Diagnostic and Production Animal Medicine, College of Veterinary Medicine, “Transmissible Gastroenteritis (TGE)” in *Swine Disease Manual.* **2019.** Retrieved from <https://vetmed.iastate.edu/vdpam/FSVD/swine/index-diseases/tge>
10. World Organisation for Animal Health. OIE Technical Factsheet, Infection with Porcine epidemic diarrhea virus. OIE. **2014**. Retrieved from <http://www.oie.int/doc/ged/D13924.PDF>
11. Bolin, S.R.; “Chapter 3. Bovine viral diarrhea virus in mixed infection” in *Polymicrobial Diseases.* Brogden, K.A.; Guthmiller, J.M., Eds. ASM Press, **2002**. Retrieved from <https://www.ncbi.nlm.nih.gov/books/NBK2491/>
12. Food and Agriculture Organization of the United Nations Secretariat of the Pacific Community, “A130 – Classical Swine Fever (hog cholera)” in *AHP Manual – Reference Guide for Animal Health Staff,* Fediaevsky, A., Ed. **2002.** Retrieved from <http://lrd.spc.int/ext/Disease_Manual_Final/a130__classical_swine_fever_hog_cholera.html>
13. World Organisation for Animal Health, “Chapter 2.8.3. Classical swine fever” in *World Organisation for Animal Health (OIE) Manual of diagnostic tests and vaccines for terrestrial animals*, OIE. **2014**, pp. 1-26. Retrieved from <http://www.oie.int/fileadmin/Home/eng/Health_standards/tahm/2.08.03_CSF.pdf>
14. World Organisation for Animal Health, “Chapter 2.3.7. Duck virus enteritis” in *World Organisation for Animal Health (OIE) Manual of diagnostic tests and vaccines for terrestrial animals*, OIE. **2012**, pp. 1-11. Retrieved from <http://www.oie.int/fileadmin/Home/eng/Health_standards/tahm/2.03.07_DVE.pdf>
15. Food and Agriculture Organization of the United Nations Secretariat of the Pacific Community, “B305 – Duck virus enteritis” in *AHP Manual – Reference Guide for Animal Health Staff,* Fediaevsky, A., **2002**. Ed. Retrieved from <http://lrd.spc.int/ext/Disease_Manual_Final/b305__duck_virus_enteritis.html#auto_top>
16. World Organisation for Animal Health, “Chapter 2.4.12. Infectious bovine rhinotracheitis/Infectious pustular vulvovaginitis” in *World Organisation for Animal Health (OIE) Manual of diagnostic tests and vaccines for terrestrial animals*. OIE, **2017**, pp. 1-19. Retrieved from <http://www.oie.int/fileadmin/Home/eng/Health_standards/tahm/2.04.12_IBR_IPV.pdf>
17. Easton, C.; Fuentealba, N.A.; Paullier, C.; Alonzo, P.; Carluccio, J.; Galosi, C.M. Immunohistochemical and molecular detection of equine herpesvirus 1 in Uruguay. *Rev sci tech Off int Epiz.* **2009** *28(3)*, 1085-1090. Retrieved from <http://www.oie.int/doc/ged/D7123.PDF>
18. World Organisation for Animal Health, “Chapter 2.5.9. Equine rhinopneumonitis (Infection with equine herpesvirus-1 and -4)” in *World Organisation for Animal Health (OIE) Manual of diagnostic tests and vaccines for terrestrial animals*, OIE. **2017**, pp. 1-13. Retrieved from <http://www.oie.int/fileadmin/Home/eng/Health_standards/tahm/2.05.09_EQUINE_RHINO.pdf>
19. Barrandeguy, M.; Vissani, A.; Lezica, F.P.; Salamone, J.; Heguy, A.; Becerra, L.; Olguin Perglione, C.; Thiry, E. Subclinical infection and periodic shedding of equid herpesvirus 3. *J. theriogenology* . **2010** *(4)*, 576-80. Retrieved from <https://www.ncbi.nlm.nih.gov/pubmed/20494427> PMID: 20494427
20. Food and Agriculture Organization of the United Nations Secretariat of the Pacific Community, “B302 – Avian infectious laryngotracheitis.” in *AHP Manual – Reference Guide for Animal Health Staff,* Fediaevsky, A., Ed. **2002**. Retrieved from <http://lrd.spc.int/ext/Disease_Manual_Final/b302__avian_infectious_laryngotracheitis.html>
21. Bagust, T.J.; Jones, R.C.; Guy, J.S. Avian infectious laryngotracheitis. *Rev sci techOff int Epiz*. **2000**, *19(2)*, 483-492. Retrieved from <http://www.oie.int/doc/ged/D9312.PDF>
22. World Organisation for Animal Health, “Chapter 2.3.13. Marek’s disease” in *World Organisation for Animal Health (OIE) Manual of diagnostic tests and vaccines for terrestrial animals*, OIE. **2017**, pp. 1-13. Retrieved from <http://www.oie.int/fileadmin/Home/fr/Health_standards/tahm/2.03.13_MAREK_DIS.pdf>
23. Payne,L.N.; Venugopal, K.; Neoplastic diseases: Marek´s disease, avian leucosis and reticuloendotheliosis. *Rev sci techOff int Epiz.* **2000** *19(2)*, 544 - 64. Retrieved from <http://www.oie.int/doc/ged/D9316.PDF>
24. Wittmann, G. Aujeszky´s disease. *Rev sci techOff int Epiz*. **1986**, *5(4)*, 959-77. Retrieved from <http://www.oie.int/doc/ged/D8544.PDF>
25. Food and Agriculture Organization of the United Nations Secretariat of the Pacific Community, “B052 – Aujeszky’s disease.” in *AHP Manual – Reference Guide for Animal Health Staff,* Fediaevsky, A., Ed. **2002.** Retrieved from <http://lrd.spc.int/ext/Disease_Manual_Final/b052__aujeszkys_disease.html>
26. Food and Agriculture Organization of the United Nations Secretariat of the Pacific Community, “A150 – Avian influenza” in *AHP Manual – Reference Guide for Animal Health Staff,* Fediaevsky,A., Ed. **2002.** Retrieved from <http://lrd.spc.int/ext/Disease_Manual_Final/a150__avian_influenza.html#auto_top>
27. Spickler, A.N.; **2009**. Avian influenza. Retrieved from <http://www.cfsph.iastate.edu/Factsheets/pdfs/highly_pathogenic_avian_influenza.pdf>
28. World Organisation for Animal Health, “Chapter 2.5.7. Equine influenza (Infection with equine influenza virus)” in *World Organisation for Animal Health (OIE) Manual of diagnostic tests and vaccines for terrestrial animals*, OIE. **2016**, pp. 1-16. Retrieved from <http://www.oie.int/fileadmin/Home/eng/Health_standards/tahm/2.05.07_EQ_INF.pdf>
29. Food and Agriculture Organization of the United Nations Secretariat of the Pacific Community, “Swine influenza” in *AHP Manual – Reference Guide for Animal Health Staff,* Fediaevsky, A., Ed. **2002.** Retrieved from <http://lrd.spc.int/ext/Disease_Manual_Final/swine_influenza.html>
30. World Organisation for Animal Health. Technical disease cards, Swine influenza. OIE. **2009**. Retrieved from <http://www.oie.int/fileadmin/Home/eng/Animal_Health_in_the_World/docs/pdf/Disease_cards/SWINE_INFLUENZA.pdf>
31. Food and Agriculture Organization of the United Nations Secretariat of the Pacific Community, “A160 – Newcastle disease” in *AHP Manual – Reference Guide for Animal Health Staff,* Fediaevsky, A., **2002.** Ed. Retrieved from <http://lrd.spc.int/ext/Disease_Manual_Final/a160__newcastle_disease.html>
32. World Organisation for Animal Health. Technical disease cards, Newcastle disease. OIE. **2013**. Retrieved from <http://www.oie.int/fileadmin/Home/eng/Animal_Health_in_the_World/docs/pdf/Disease_cards/NEWCASTLE_DISEASE.pdf>
33. World Organisation for Animal Health. Technical disease cards, Peste des petis ruminants. OIE. **2013**. Retrieved from <http://www.oie.int/fileadmin/Home/eng/Animal_Health_in_the_World/docs/pdf/Disease_cards/PESTE_DES_PETITS_RUMINANTS.pdf>
34. World Organisation for Animal Health. Technical disease cards, Rinderpest. OIE. **2013**. Retrieved from <http://www.oie.int/fileadmin/Home/eng/Animal_Health_in_the_World/docs/pdf/Disease_cards/RINDERPEST.pdf>
35. Food and Agriculture Organization of the United Nations Secretariat of the Pacific Community, “C853- Avian encephalomyelitis” in *AHP Manual – Reference Guide for Animal Health Staff,* Fediaevsky, A., Ed. **2002.** Retrieved from <http://lrd.spc.int/ext/Disease_Manual_Final/c853__avian_encephalomyelitis.html>
36. Food and Agriculture Organization of the United Nations Secretariat of the Pacific Community, “B256 – Enterovirus encephalomyelitis (previous Teschen disease)” in *AHP Manual – Reference Guide for Animal Health Staff,* Fediaevsky, A., Ed.**2002**. Retrieved from <http://lrd.spc.int/ext/Disease_Manual_Final/b256__enterovirus_encephalomyelitis.html>
37. World Organisation for Animal Health. Technical disease cards, Foot and mouth disease. OIE. **2013**. Retrieved from <http://www.oie.int/fileadmin/Home/eng/Animal_Health_in_the_World/docs/pdf/Disease_cards/FOOT_AND_MOUTH_DISEASE.pdf>
38. World Organisation for Animal Health, “Chapter 2.1.8. Foot and mouth disease” in *World Organisation for Animal Health (OIE) Manual of diagnostic tests and vaccines for terrestrial animals*. OIE. **2017**, pp. 1-32. Retrieved from <http://www.oie.int/fileadmin/Home/eng/Health_standards/tahm/2.01.08_FMD.pdf>
39. World Organisation for Animal Health. Technical disease cards, Swine vesicular disease. OIE. **2013**. Retrieved from <http://www.oie.int/fileadmin/Home/eng/Animal_Health_in_the_World/docs/pdf/Disease_cards/SWINE_VESICULAR_DISEASE.pdf>
40. Food and Agriculture Organization of the United Nations Secretariat of the Pacific Community, “A030 – Swine vesicular disease” in *AHP Manual – Reference Guide for Animal Health Staff,* Fediaevsky,A., Ed. **2002.** Retrieved from <http://lrd.spc.int/ext/Disease_Manual_Final/a030__swine_vesicular_disease.html>
41. Food and Agriculture Organization of the United Nations Secretariat of the Pacific Community, “B307 – Fowl pox” in *AHP Manual – Reference Guide for Animal Health Staff,* Fediaevsky, A., Ed. **2002.** Retrieved from <http://lrd.spc.int/ext/Disease_Manual_Final/b307__fowl_pox.html>
42. World Organisation for Animal Health. Technical disease cards, Sheep pox and goat pox (OIE, 2013). Retrieved from <http://www.oie.int/fileadmin/Home/eng/Animal_Health_in_the_World/docs/pdf/Disease_cards/SHEEP_GOAT_POX.pdf>
43. Food and Agriculture Organization of the United Nations Secretariat of the Pacific Community, “A100 – Sheep and goat pox” in *AHP Manual – Reference Guide for Animal Health Staff,* Fediaevsky, A., Ed.**2002.**  Retrieved from <http://lrd.spc.int/ext/Disease_Manual_Final/a100__sheep_pox_and_goat_pox.html>
44. World Organisation for Animal Health. Technical disease cards, Lumpy skin disease. OIE. **2017**. Retrieved from <http://www.oie.int/fileadmin/Home/eng/Animal_Health_in_the_World/docs/pdf/Disease_cards/LUMPY_SKIN_DISEASE_FINAL.pdf>
45. Spickler, A.N.; **2015**. Contagious ecthyma. Retrieved from <http://www.cfsph.iastate.edu/Factsheets/pdfs/contagious_ecthyma.pdf>
46. World Organisation for Animal Health. Technical disease cards, Bluetongue. OIE. **2013**. Retrieved from <http://www.oie.int/fileadmin/Home/eng/Animal_Health_in_the_World/docs/pdf/Disease_cards/BLUETONGUE.pdf>
47. van der Sluijs, M.T.W.; Schroer-Joosten, D.P.H.; Fid-Fourkour, A.; Vrijenhoek, M.P.H.; Debyser, I.; Moulin, V.; Moormann, R.J.M.; de Smit, A.J. Transplacental transmission of bluetongue virus serotype 1 and serotype 8 in sheep: virological and pathological findings. *PLoS ONE*. **2013,** *8(12*). Retrieved from <https://doi.org/10.1371/journal.pone.0081429>
48. See 23 above.
49. Food and Agriculture Organization of the United Nations Secretariat of the Pacific Community, “B108 – Enzootic bovine leukosis” in *AHP Manual – Reference Guide for Animal Health Staff,* Fediaevsky, A., Ed. **2002.** Retrieved from <http://lrd.spc.int/ext/Disease_Manual_Final/b108__enzootic_bovine_leukosis.html>
50. European Food Safety Authority, EFSA Scientific opinion Enzootic bovine leukosis – EFSA Panel on animal health and welfare (AHAW). *J EFS.* **2015**, *13*. Retrieved from <https://efsa.onlinelibrary.wiley.com/doi/epdf/10.2903/j.efsa.2015.4188>
51. Food and Agriculture Organization of the United Nations Secretariat of the Pacific Community, “B153 – Caprine arthritis/encephalitis” in *AHP Manual – Reference Guide for Animal Health Staff,* Fediaevsky, A., Ed. **2002.** Retrieved from <http://lrd.spc.int/ext/Disease_Manual_Final/b153__caprine_arthritisencephalitis.html>
52. Spickler, A.R.; Small ruminant lentiviruses. **2015.** Retrieved from <http://www.cfsph.iastate.edu/Factsheets/pdfs/maedi_visna_and_caprine_arthritis_encephalitis.pdf>
53. Food and Agriculture Organization of the United Nations Secretariat of the Pacific Community, “B205 – Equine infectious anemia” in *AHP Manual – Reference Guide for Animal Health Staff,* Fediaevsky, A., Ed.**2002.** Retrieved from <http://lrd.spc.int/ext/Disease_Manual_Final/b205__equine_infectious_anaemia.html>
54. The Center for Food Security and Public Health – Iowa State University, Technical Factsheet Ovine pulmonary adenomatosis*.* **2009*.*** Retrieved from <http://www.cfsph.iastate.edu/Factsheets/pdfs/ovine_pulmonary_adenomatosis.pdf>
55. Caporale, M.; Centorame, P.; Giovannini, A.; Sacchini, F.; Di Ventura, M.; De las Heras, M.; Palmarini, M. Infection of lung epithelial cells and induction of pulmonary adenocarcinoma is not the most common outcome of naturally occurring JSRV infection during the commercial lifespan of sheep. *Virology*. **2005**, *338(1)*, 144-153. <https://www.ncbi.nlm.nih.gov/pubmed/15950254> PMID: 15950254
56. Food and Agriculture Organization of the United Nations Secretariat of the Pacific Community, “B157 - Ovine pulmonary adenomatosis” in *AHP Manual – Reference Guide for Animal Health Staff,* Fediaevsky, A., Ed. **2002.** Retrieved from <http://lrd.spc.int/ext/Disease_Manual_Final/b157_ovine_pulmonary_adenomatosis.html>
57. Food and Agriculture Organization of the United Nations Secretariat of the Pacific Community, “B161 – Maedi-Visna” in *AHP Manual – Reference Guide for Animal Health Staff,* Fediaevsky, A., Ed. **2002.** Retrieved from <http://lrd.spc.int/ext/Disease_Manual_Final/b161__maedivisna.html>
