# Supplementary material for: Animal Virus Ecology and Evolution Are Shaped by the Virus Host-Body Infiltration and Colonization Pattern
Source: Pathogens. 2019 May 25;8(2):72. doi: 10.3390/pathogens8020072 (PMC6631033; doi:10.3390/pathogens8020072)
Supplement: Supplementary file 1 [file pathogens-08-00072-s001.zip › SUPPLEMENTARY MATERIALS Figure S1d.docx]

# Supplementary Materials

Figure S1d: Outer- to inner-body line-up of 36 viruses by organ system and combination of organ systems.

Respiratory viruses

The equine influenza virus (EIV) was ranked as least infiltrative among the two, strictly respiratory, influenza viruses in the study. In horses, the virus causes a transient infection resulting in a dry cough usually abating in a few days. Virus transmission is mainly via aerosols. Also fomites may play a role.

The swine influenza virus (SIV) infection in pigs involves significant mucus production, translating in coughing and sneezing, and enhancing virus transmission also on the basis of direct contact. The infection lasts about six days while virus shedding may continue for up to a month. The virus resists low temperatures; epizootics mainly occur during the winter.

Acute viruses of both respiratory and alimentary tract

Among the acute viruses infecting both the respiratory and the alimentary tract the rinderpest virus (RPV) was ranked first. Clinical signs in cattle and buffaloes comprise oculonasal discharge, excessive salivation and diarrhea. The virus becomes transmitted during close, muzzle-to-muzzle contact. The virus is mainly excreted during the first week of infection. Fomites are not a viable means of transmission. There are no carriers.

Next, infection by the closely related peste des petits ruminants virus (PPRV) in sheep and goats results in significant respiratory discharge and diarrhea. The infection takes up to 14 days and there are no carriers. Virus transmission is on the basis of direct contact or via aerosols.

Next, in poultry the avian influenza virus (AIV) causes respiratory and alimentary tract infections, variable in severity. Highly pathogenic avian influenza in chicken usually results in per-acute death. Mild chicken viruses are also common. Virus transmission is on the basis of aerosols, through direct contact or involving fomites, contaminated feed stuffs or water. The virus is excreted for about a week and survives up to 32 days in the environment.

Acute plus persistent viruses of both respiratory and alimentary tract

The avian paramyxovirus type 1 (APMV-1) causes respiratory, gastrointestinal, and nervous infections in poultry. Also clinically normal birds may carry the virus. Transmission is both airborne and fecal-oral. The virus survives well at ambient temperatures in feces.

Next, the infectious bronchitis virus (IBV) in poultry is shed in tracheal mucus and in feces. The infection is mostly resolved within 14 days. Latent infection and erratic virus shedding may occur.

Alimentary tract virus

Among the viruses colonizing the alimentary tract, the porcine epidemic diarrhea virus (PEDV) causes diarrhea lasting six to 35 days after the onset of clinical signs. Transmission is fecal-oral, involves fomites, feed, or people. The virus survives for weeks outside the body.

**Viruses infecting mucosae and skin**

Among the viruses colonizing mucosae and skin the sheep and goat pox virus (SGPV) was ranked first. In small ruminants the pox lesions take weeks to heal. Clinical signs comprise oculonasal discharge. Virus transmission is direct or indirect. The virus is extremely robust as it may remain infective in scabs for two months or for six months in the environment.

Next, the contagious pustular dermatitis virus (CPDV) in small ruminants causes lesions on mouth, muzzle, udder, and feet. Most infections heal in three to six weeks. Virus transmission is mainly through direct contact. After recovery, virus on wool and hide remains infective for up to one month.

Next, the fowlpox virus (FWPV) in chickens and turkeys causes dry skin lesions or diphteritic, wet nodules in mouth and trachea, resulting in a protracted course of infection. The virus slowly spreads in poultry flocks. The virus transmits during close contact or through skin abrasions; the virus is abundantly present in the skin lesions. Also mechanical transmission by biting flies is possible.

**Epithelial viruses featuring a systemic component**

The porcine enterovirus encephalomyelitis virus (PEV1) in pigs is excreted in feces and in oral secretions. Recovered animals may continue to shed virus for up to seven weeks. Virus transmission is fecal-oral. The virus may survive in the environment for three months.

The swine vesicular disease virus (SVDV) in pigs causes vesicles on the feet, lower limbs, and snout. Virus excretion from nose and mouth usually stops within two weeks. In addition, there is evidence for a systemic infection component. Virus shedding in feces may last up to three months. The virus spreads mainly through direct contact. Also swill feeding may start an outbreak. The virus is environmentally resistant.

**Epithelial viruses latently present also in peripheral nerves plus ganglia**

Among the epithelial viruses which are also latently present in peripheral nerves and ganglia, equine herpesvirus-3 (EHV-3) was ranked first for causing non-invasive lesions on the external genitalia, and which tend to heal in ten to fourteen days. Transmission is on the basis of sexual or genitonasal contact. The virus is environmentally labile.

Next, the bovine herpesvirus-1 (BHV-1), infectious bovine rhinotracheitis or infectious pustular vulvovaginitis/infectious pustular balanoposthitis virus in cattle and buffalo typically infects either the respiratory tract or the external genitalia. The virus may be secreted from vagina or prepuce. Virus may also be present in semen. In addition, venereal infection may trigger abortion or a fatal systemic infection in new-borne calves. The venereal infection lasts five to ten days while virus shedding may continue two to sixteen days after infection.

Next, the gallid herpesvirus-1 (GaHV-1) or avian laryngotracheitis virus infection results in the production of bloodstained mucus along the length of the trachea. Virus is latently present in the trigeminal ganglion from where it may reactivate and trigger a recurrent respiratory infection. Virus transmission is via aerosol, through direct contact and fomites. The virus may persist for months in tracheal mucus or carcasses.

Next, the duck enteritis virus (DEV) in ducks, geese and swans is responsible for acute diarrhea. Clinical signs comprise oculonasal discharge. Virus transmission is through direct contact and fomites. Fecal contamination of eggshells may transmit the virus to hatching chicks. Latently infected birds may continue to shed the virus in feces.

**Systemic viruses involving epithelia, nerves plus ganglia, and reproductive system**

Among the systemic viruses infecting the epithelia, nerves and ganglia, and the reproductive system the equine herpesvirus-1 (EHV-1) or equine rhino-pneumonitis virus was ranked first. The virus causes respiratory, neurological and reproductive disorders. The respiratory infection is usually mild and subsides within two weeks. Abortion tends to occur during the third semester. Abortion storms may result from subsequent lateral, airborne virus transmission. Infection around the time of birth may result in a weak, leukopenic foal which dies after a few days.

Next, the swine herpesvirus-1 (SHV-1) or Aujeszky disease virus in pigs may also cause neurological, reproductive, and respiratory disorders. Haphazard abortion, stillbirth and birth of infected piglets occur. Virus reactivation from nerves and ganglia may translate in virus shedding in vaginal secretions, semen and colostrum. Hence, virus transmission is via aerosols, venereal, congenital, and lactogenic. Also swill feeding may kick-start an outbreak; the virus survives up to five weeks in pig meat.

## Systemic virus involving epithelia, nerves plus ganglia, and immune system

The gallid herpesvirus-2 (GaHV-2) or Marek disease virus in poultry is responsible for visceral lymphomas and neuropathological disorder. Proliferation of lymphoid cells is typically followed by a latency phase, immunosuppression, and formation of neoplasms. Virus is shed from feather follicles for life. Virus transmission results from inhalation of infected dust. The virus remains infective for months to years.

## Systemic viruses involving epithelia and reproductive system, haphazardly

The transmissible gastroenteritis virus (TGEV) in pigs causes vomiting and profuse diarrhea. Mortality is highest in young piglets not protected by colostral antibodies. In adults, recovery takes five to ten days. Carrier sows may shed virus in feces or milk. Transmission is via aerosols, through direct contact, fomites or vertically via the milk. The virus resists low temperatures; epizootics occur mainly during the winter.

Ranked next was the lumpy skin disease virus (LSDV) in cattle causes pox lesions on skin and mucosae and is shed in milk and semen. Pregnant cows may abort. Virus shedding maybe prolonged as acute infection translates in a carrier stage. Virus transmission is mainly by biting flies, mechanically.

Next, the foot and mouth disease virus (FMDV) in cloven-hoofed animals may during incubation be shed in milk and semen. Upon onset of clinical signs virus may be present in breath, saliva, feces, and urine. Vesicles are found on the feet, buccal mucosa, and teats. The carrier stage may last up to six months. Transmission is via aerosols, through direct contact, fomites, or seminal. In pigs, swill feeding may play a role. The virus is environmentally resistant.

**Systemic viruses involving epithelia and reproductive system, in an orderly fashion**

Next, the avian encephalitis virus (AEV) in poultry is responsible for congenital infections in chickens. The virus is also neurotropic and enterotropic. After hatching the infected chicks may shed virus in feces and so transmit virus to susceptible chicks. Virus may be shed in feces for several weeks. In adults, carriers may result.

Next, the equine arteritis virus (EAV) virus usually causes a mild infection. Signs in horses may include nasal discharge, rhinitis, conjunctivitis, edema, dyspnea, diarrhea, and abortion. Stallions tend to become long term carriers and continue to shed virus in semen. Transmission is by aerosols, seminal and, incidentally, congenital.

Next, the classical swine fever virus (CSFV) infection in carrier sows may typically result in the birth to weak or also sub-clinically infected piglets. Clinical infections vary from severe per-acute to mild and chronic forms. Virus may be present in oculonasal discharge, blood, feces and semen. Hence, transmission is fecal-oral, through direct contact, fomites, venereal or congenital. Also swill feeding may trigger an outbreak; the virus is fairly resistant.

**Systemic viruses involving epithelia and reproductive system, and featuring congenital plus lactogenic transmission**

The porcine reproductive and respiratory syndrome virus (PRRSV) causes respiratory disorders in young piglets and reproductive failure in sows. Virus transmission may be both congenital and lactogenic. In addition, the virus may spread via the respiratory route, through direct contact, swill feeding or venereally. Hence, virus may be present in saliva, feces, urine, semen, and colostrum. Persistently infected sows may abort or, also, give birth to weak or apparently healthy infected piglets.

## Next, for the bovine viral diarrhea virus (BVDV) congenital transmission plays a central role in the virus transmission cycle. Virus may also be present in saliva, feces, blood, urine, semen, colostrum, and aborted materials. The infections in cattle vary from subclinical to severe to fatal. Signs may include ulceration of the alimentary mucosa, salivation, respiratory signs, leucopenia, abortion, and congenital anomalies. Acutely infected cattle shed the virus for a short period. Intrauterine infection may result in a persistently infected calf. The virus is environmentally sensitive.

**Systemic virus involving epithelia and immune system**

The infectious bursal disease virus (IBDV) is an immune-suppressive enteric birnavirus of domestic fowl. The disease is most common in three to six weeks old birds. The virulence level varies. The cloacal bursa is enlarged. The virus is shed in feces. Recovered birds may carry the virus for long periods of time. The virus is environmentally resistant and readily transmitted mechanically among farms by people, equipment and vehicles.

## Systemic virus involving epithelia, reproductive, immune plus circulatory systems

The avian leukosis virus (ALV) in domestic fowl causes a mostly subclinical infection. Chicken with subclinical disease usually shed virus into the albumen or yolk of eggs. Congenitally infected chicks remain viremic for life. Also leukemia-like infections result, or a proliferative disease of the hemopoietic system, clonal malignancies of the bursal-dependent lymphoid system, or myeloid leukosis. Clinical signs include weakness, diarrhea, dehydration and emaciation. Virus may be present in feces, saliva, and desquamated skin. Virus survival outside the host body is restricted to a few hours.

**Systemic viruses involving the reproductive system and featuring restricted epithelial transmission**

The Maedi-Visna virus (MVV) and the caprine arthritis-encephalitis virus (CAEV) are closely related lentiviruses of sheep and goats and feature lactogenic virus transmission. Most animal become infected early in life from drinking infected colostrum or milk. Most infections are subclinical and for life. In sheep, progressive dyspnea or neurological signs may be observed. Encephalomyelitis and poly-arthritis tend to develop in goats. Once clinical signs appear the disease is progressive and usually fatal. Virus may be present in respiratory secretions, feces, semen, colostrum and milk. Virus transmission resulting from direct contact is uncommon; the virus is labile.

**Systemic viruses involving reproductive, immune plus circulatory systems, and featuring restricted epithelial transmission**

Next, the jaagsiekte sheep retrovirus (JSRV) causes clinical signs only in the minority of sheep that develop pulmonary adenomatosis. The virus is more readily detectable in peripheral blood leucocytes and lymphoid organs than in the lungs. Incubation varies from six months to three years. Once a lung tumor develops, the signs are progressive and comprise emaciation, weight loss and respiratory compromise, ending in severe dyspnea and death. Transmission is via milk and colostrum or by the respiratory route, probably via aerosols or droplets. The virus is environmentally labile.

## Systemic viruses involving reproductive, immune plus circulatory systems, and featuring an absence of epithelial transmission

Among the viruses involving the reproductive, immune and circulatory systems the bovine leukemia virus (BLV) is ranked first. The virus causes lymphomas in two to five percent of infected animals, predominantly adult cattle older than three to five years. The main transmission is perinatal from cow to calf, via colostrum and milk. Also important are trans-placental transmission, mechanical transmission by bloodsucking insects, iatrogenic and seminal transmission. The virus is environmentally labile.

Next, the equine infectious anemia virus (EIAV) in horses and other equids may cause a debilitating disease. Subclinical and mild infections are the most common. Bloodsucking flies, in particular of the Tabanidae, transmit the virus mechanically. Also intrauterine and transmission via colostrum or milk occur. The virus is environmentally labile.

**Systemic virus involving the reproductive system, blood circulation, plus a hematophagous arthropod, and featuring an absence of epithelial transmission**

Finally, only partially overlapping with the above is the transmission ecology of the bluetongue virus (BTV). This reovirus infects sheep and other ruminants, domestic and wild, plus undergoes an infection cycle in *Culicoides* midges. After six to eight days replication in the salivary glands midges remain infective for life. In sheep most infections are in-apparent or transient. Also seminal and trans-placental transmission may occur. The epithelial infection does not contribute to the virus transmission success. The virus is environmentally labile.
