# Supplementary material for: Animal Virus Ecology and Evolution Are Shaped by the Virus Host-Body Infiltration and Colonization Pattern
Source: Pathogens. 2019 May 25;8(2):72. doi: 10.3390/pathogens8020072 (PMC6631033; doi:10.3390/pathogens8020072)
Supplement: Supplementary file 1 [file pathogens-08-00072-s001.zip › SUPPLEMENTARY MATERIALS Figure S1b.docx]

# Supplementary Materials

**Figure S1b:**  **The scores allocated to the 36 viruses for four ecological variables and the virus host range.**

| **Virus name in full**  **and abbreviated** | **One-to-three**  **virus host-body**  **infiltration score,**  **as indicated by the organ systems involved in infection-transmission**  **1. epithelia**  **2. epithelia plus**  **internal organs**  **3. internal organs** | **One-to-four**  **virus host-body**  **infiltration score,**  **as indicated by the**  **organ systems involved**  **in infection-transmission**  **1. strictly epithelia**  **2. primarily epithelia**  **3. epithelia plus**  **internal organs**  **4. primarily**  **internal organs** | **Length of**  **the infection period**  **score**  **1. acute**  **2. acute plus**  **persistent**  **3. persistent** | **Infection severity**  **level**  **score**  **CF = case**  **fatality**  **1. CF% < 1**  **2. CF% 1-10**  **3. CF% > 10** |  | **Virus environmental survival rate score**  **1 < 3 days**  **2. 3-10 days**  **3. > 10 days** | **Host range** |  |
| --- | --- | --- | --- | --- | --- | --- | --- | --- |
|  |  |  |  |  |  |  |  |  |
| equine arteritis virus (EAV) | 2 | 3 | 2 | 1 |  | 1 | Family  (Equidae) |  |
| porcine reproductive and respiratory syndrome virus (PRRSV) | 2 | 3 | 2 | 3 |  | 2 | Species  *(Sus scrofa)* |  |
| avibirnavirus (IBDV); infectious bursal disease virus | 2 | 3 | 2 | 3 |  | 3 | Class  *(Aves)* |  |
| gammacoronavirus infectious bronchitis virus (IBV) | 1 | 1 | 2 | 2 |  | 2 | Family (Phasianidae) |  |
| transmissible gastroenteritis virus (TGEV) | 2 | 3 | 2 | 2 |  | 2 | Species  *(Sus scrofa)* |  |
| porcine epidemic diarrhea virus (PEDV) | 1 | 1 | 2 | 3 |  | 3 | Species  *(Sus scrofa)* |  |
| bovine viral diarrheavirus (BVDV) | 2 | 3 | 2 | 2 |  | 2 | Order (Artiodactyla) |  |
| classical swine fever virus (CSFV) | 2 | 3 | 2 | 3 |  | 2 | Species  *(Sus scrofa)* |  |
| anatid herpes-1 (DEV); duck virus enteritis virus | 2 | 2 | 2 | 3 |  | 2 | Order (Anseriformes) |  |
| bovine herpesvirus-1 (BHV-1) | 2 | 2 | 2 | 1 |  | 3 | Order (Artiodactyla) |  |
| equine herpesvirus-1 (EHV-1) | 2 | 3 | 2 | 1 |  | 2 | Family  (Equidae) |  |
| equine herpesvirus-3 (EHV-3) | 2 | 2 | 2 | 1 |  | 1 | Species *(Equus ferus caballus)* |  |
| gallid herpesvirus-1 (GaHV-1) | 2 | 2 | 2 | 2 |  | 3 | Family (Phasianidae) |  |
| gallid herpesvirus-2 (GaHV-2) | 2 | 3 | 3 | 2 |  | 3 | Species *(Gallus g. domesticus)* |  |
| suid herpesvirus-1 (SHV-1) | 2 | 3 | 2 | 2 |  | 3 | Species  *(Sus scrofa)* |  |
| avian influenza virus (AIV) | 1 | 1 | 1 | 3 |  | 3 | Class (Aves) |  |
| equine influenza virus (EIV) | 1 | 1 | 1 | 1 |  | 1 | Family (Equidae) |  |
| swine influenza virus (SIV) | 1 | 1 | 2 | 2 |  | 2 | Species  *(Sus scrofa)* |  |
| avian paramyxovirus type 1 (APMV-1) | 1 | 1 | 2 | 3 |  | 3 | Class  (Aves) |  |
| peste des petits ruminants virus (PPRV) | 1 | 1 | 1 | 3 |  | 2 | Order (Artiodactyla) |  |
| Rinderpest virus (RPV) | 1 | 1 | 1 | 3 |  | 1 | Order (Artiodactyla) |  |
| avian encephalitis virus (AEV) | 2 | 3 | 2 | 2 |  | 3 | Order (Galliformes) |  |
| enterovirus encephalomyelitis (PEV1) | 1 | 2 | 2 | 2 |  | 3 | Species  *(Sus scrofa)* |  |
| foot and mouth disease virus (FMDV) | 2 | 3 | 2 | 2 |  | 3 | Order (Artiodactyla) |  |
| swine vesicular disease virus (SVDV) | 1 | 2 | 2 | 1 |  | 3 | Species  *(Sus scrofa)* |  |
| avipoxvirus (FWPV); fowlpox virus | 1 | 1 | 2 | 2 |  | 3 | Order (Galliformes) |  |
| capripoxvirus (SGPV); sheep and goat pox virus | 1 | 1 | 2 | 2 |  | 3 | Subfamily (Caprinae) |  |
| lumpy skin disease virus (LSDV) | 2 | 3 | 3 | 1 |  | 3 | Order (Artiodactyla) |  |
| parapoxvirus (CPDV); contagious pustular disease virus | 1 | 1 | 2 | 1 |  | 3 | Subfamily (Caprinae) |  |
| bluetongue virus (BTV) | 3 | 4 | 2 | 1 |  | 1 | Order (Artiodactyla) |  |
| avian leukosis virus (ALV) | 2 | 3 | 3 | 2 |  | 1 | Class  (Aves) |  |
| bovine leukemia virus (BLV) | 3 | 4 | 3 | 2 |  | 1 | Subfamily (Bovinae) |  |
| caprine arthritis-encephalitis virus (CAEV) | 2 | 4 | 3 | 1 |  | 1 | Subfamily (Caprinae) |  |
| equine infectious anemia virus (EIAV) | 3 | 4 | 3 | 1 |  | 1 | Family  (Equidae) |  |
| jaagsiekte, sheep retrovirus (JSRV) | 2 | 4 | 3 | 2 |  | 1 | Species *(Ovis aries)* |  |
| Maedi-Visna virus (MVV) | 2 | 4 | 3 | 1 |  | 1 | Subfamily (Caprinae) |  |
