# Supplementary material for: Animal Virus Ecology and Evolution Are Shaped by the Virus Host-Body Infiltration and Colonization Pattern
Source: Pathogens. 2019 May 25;8(2):72. doi: 10.3390/pathogens8020072 (PMC6631033; doi:10.3390/pathogens8020072)
Supplement: Supplementary file 1 [file pathogens-08-00072-s001.zip › SUPPLEMENTARY MATERIALS Figure S1a.docx]

# Supplementary Materials

**Figure S1a: Details on the 36 viruses, with a brief summary on the virus transmission ecology.**

| **Virus family** | **Genomic architecture** | **Virus name**  **in full and**  **abbreviated** | **Name given to**  **infection or**  **disease** |  | **Brief summary on the virus transmission ecology,**  **with references to the primary livestock host, the virus organ system tropism, the length of the infection and shedding period, the infection severity level, the transmission modes, and the virus environmental survival rate.**  **In brackets the literature sources as shown in**  **Supplementary Materials Figure S1c.** |  |
| --- | --- | --- | --- | --- | --- | --- |
|  |  |  |  |  |  |  |
| *Arteriviridae* | (+)ssRNA | *equine arteritis virus (EAV)* | equine viral arteritis, epizootic lymphangitis, pinkeye |  | The virus causes a mostly mild, systemic infection in equids, chiefly horses. Clinical signs include nasal discharge, rhinitis, conjunctivitis, edema, dyspnea, diarrhea, and abortion. Stallions may become long term carriers and transmit virus in semen. Mares infected in late pregnancy may give birth to infected foals. Virus transmission is by aerosols, venereal and, incidentally, congenital. The virus remains viable for 2 to 3 days at 37-38 degrees Celsius. (1, 2) |  |
|  |  | *porcine reproductive and respiratory syndrome virus (PRRSV)* | porcine reproductive and respiratory syndrome |  | The virus causes reproductive failure in sows and respiratory problems in piglets and growing pigs. High mortality in piglets and of ten percent or more in sows. The reproductive failure comprises infertility, abortion, stillbirth, birth of weak offspring or, also, of apparently healthy carriers. The virus may be present in saliva, feces, urine, semen, and colostrum. Virus transmission may be airborne, through direct contact, venereal, intrauterine, congenital, lactogenic, involve fomites, vehicles, persons, or result from swill feeding. (3, 4) |  |
| *Birnaviridae* | dsRNA | *avibirnavirus; infectious bursal disease virus*  *(IBDV)* | infectious bursal disease, Gumboro disease |  | The enteric virus infiltrates and establishes in the cloacal bursa of Fabricius and triggers immunosuppression in chicken. Mortality rates of up to 25 percent in broilers and 60 percent in layers may occur. Carriers may shed virus in feces for extended periods of time. Virus transmission may involve contaminated water, feed, poultry house litter, fomites, vehicles, or persons. The virus is environmentally resistant. (5, 6) |  |
| *Coronaviridae* | (+)ssRNA | *gammacoronavirus infectious bronchitis virus*  *(IBV)* | avian infectious bronchitis |  | The virus causes respiratory and alimentary tract infections in chicken. Virus is present in accumulated tracheal mucus and in feces, in the acute and recovery phase of the infection respectively. Mortality may be high in young chicken. Infection is mostly resolved within 14 days. Also latent infection and erratic virus shedding for a prolonged period of time, in both feces and aerosol, is possible. Rapid airborne spread. Also a fecal-oral cycle or transmission through contaminated feed or water. (7, 8) |  |
|  |  | *transmissible gastroenteritis virus (TGEV)* | transmissible gastroenteritis |  | The virus causes vomiting and profuse diarrhea in pigs. Carrier animals often are a source of exposure to TGE virus. Mortality may be high in piglets not protected by colostral antibodies. Recovery usually occurs in 5-10 days. The infection spreads rapidly by aerosol or contact exposure. Also fomites play a role. Infected sows can transmit virus in their milk or feces to their piglets. The virus resists lower temperatures and epizootics are more common during the winter. (9) |  |
|  |  | *porcine epidemic diarrhea virus*  *(PEDV)* | porcine epidemic diarrhea |  | The virus causes a watery diarrhea in pigs. The infection can last 6-35 days after the onset of clinical signs. The virus is transmitted through the fecal-oral cycle, contaminated feed, vehicles, people, and fomites. In moist conditions and at ambient temperatures the virus may survive for several weeks outside the host body. The virus survives for four weeks in wet feed mixture at 25 degrees Celsius. (10) |  |
| *Flaviviridae* | (+)ssRNA | *bovine viral diarrhea*  *virus*  *(BVDV)* | bovine viral diarrhea |  | The virus is responsible for congenital, persistent infections, mucosal disease, and acute diarrhea in cattle and buffaloes. Congenital infection plays a central role in virus transmission. Virus may be present in oculonasal discharges, saliva, uterine fluids, amniotic fluids, blood, urine, as well as feces, semen, embryos, aborted fetuses, placentas, and colostrum. Virus transmission is through direct contact and fomites or involving various bodily excretions and secretions, including via colostrum. The virus is stable at temperatures below ten degrees Celsius. (11) |  |
|  |  | *classical swine fever virus*  *(CSFV)* | classical swine fever |  | The virus causes infections in pigs that vary from severe peracute to mild and chronic. Signs include oculonasal discharges, hemorrhages, diarrhea, and abortion. The virus is excreted in various bodily fluids, including in blood. The virus may be shed also in semen. Transmission may result from direct contact, venereal transmission, involve fomites, vehicles, persons, or result from swill feeding. Also congenital infection occurs, resulting in weak offspring or, also, apparently healthy carriers. The virus is moderately fragile. Survives 7-15 days at 37 degrees Celsius. (12, 13) |  |
| *Herpesviridae* | dsDNA | *anatid herpes-1; duck enteritis virus*  *(DEV)* | duck virus enteritis |  | The virus causes an acute, severe and systemic infection in ducks, geese and swans. Signs include watery diarrhea and oculonasal discharges. Horizontal transmission is through direct contact, contaminated water or fomites. Virus shed on the eggshell surface may form a source of vertical transmission. Latently infected birds may continue to shed the virus in feces. (14, 15) |  |
|  |  | *bovine herpesvirus-1*  *(BHV-1)* | infectious bovine rhinotracheitis /  infectious pustular vulvovaginitis  IBR/IPV |  | The virus causes respiratory disease in cattle and buffaloes characterized by rhinitis or infection of the external genitalia, with pustular vulvovaginitis and balanoposthitis. In cows the virus may trigger abortion. Usually spontaneous recovery occurs. Uncomplicated infections last 5-10 days. The virus may also be shed in semen. The virus is fairly resistant to environmental influences (16) |  |
|  |  | *equine herpesvirus-1*  *(EHV-1)* | equine herpesvirus, myelo-encephalopathy, epidemic abortion |  | The virus causes a usually mild respiratory disease in horses subsiding within two weeks. Diverse clinical presentations. Also abortion, neonatal foal death, or neurological disorder may occur. Abortion in latently infected mares usually occurs in the third semester. After abortion mares typically produce normal foals in subsequent pregnancies. Occasionally, an abortion storm may occur, involving lateral, airborne virus transmission. Infection around the time of parturition may result in the birth of a weak, leukopenic foal that dies after a few days. Airborne, trans-placental and congenital transmission. (17, 18) |  |
|  |  | *equine herpesvirus-3*  *(EHV-3)* | equine coital exanthema |  | Benign venereal disease of horses. The virus causes coital exanthema and mucocutaneous lesions on the external genitalia of mares and stallions. The pock-like lesions are benign and healing takes 10-14 days. Apart from venereal transmission, infection may result from genitonasal contact. The virus is environmentally labile. Epidemiological data support virus latency in peripheral nerves and ganglia. (19) |  |
|  |  | *gallid herpesvirus-1*  *(GaHV-1)* | avian infectious laryngotracheitis |  | The virus causes an acute, very contagious respiratory disease in chicken and turkeys. Signs may include bloodstained mucus, along the length of the trachea. Transmission is through aerosols, direct contact, or fomites. The virus latently establishes in the trigeminal ganglion from where it may reactivate. Virus infectivity can persist for months in tracheal mucus or carcasses. (20) |  |
|  |  | *gallid herpesvirus-2*  *(GaHV-2)* | Marek disease |  | The virus causes a lymphomatous and neuropathic disease mainly of domestic fowl. Gross pathology comprises major enlargement of peripheral nerves, and visceral lymphomas. After inhaling infected dust the infection may start with a proliferation of lymphoid cells, followed by a latency phase, immunosuppression, and formation of neoplasms. Infection may be lifelong.Transmitted horizontally only. The virus is shed from feather follicles for life. Virus presence in dust presents an environmental repository. After inhaling infected dust the infection may start with a proliferation of lymphoid cells, followed by a latency phase, immunosuppression, and formation of neoplasms. The virus can survive in the environment for months to years. (21, 22, 23) |  |
|  |  | *suid herpesvirus-1 (SHV-1)* | Aujeszky disease, pseudorabies |  | Aujeszky’s disease is an important disease in pigs characterized by neurological, reproductive, and respiratory disorders. Pigs less than two weeks old usually die. In sows the infections varies from early death and resorption of fetuses to middle pregnancy abortion of mummified fetuses, to stillbirth, to birth of infected piglets. The principal mode of transmission is via the respiratory route. Venereal, congenital, and lactogenic transmission are possible. The virus can survive for up to three weeks outside the pig. Also swill feeding may start an outbreak; the virus survives up to five weeks in pig meat. Virus reactivation may result in virus shedding in semen, colostrum, and vaginal secretions. (24, 25) |  |
| *Orthomyxoviridae* | (-)ssRNA | *avian influenza virus*  *(AIV)* | avian influenza |  | In poultry the virus infects the respiratory and the alimentary tract. Highly pathogenic avian influenza causes per-acute death. Also a systemic infection and central nervous system involvement may be observed. Mortality may be up to 100 percent. Chicken typically excrete low pathogenic avian influenza viruses for a week. Aerosols, direct contact, fomites, feed and water may play a role in virus transmission. The virus may remain viable for up to 32 days in feces and water. (26, 27) |  |
|  |  | *equine influenza virus*  *(EIV)* | equine influenza |  | The virus causes an upper respiratory tract infection in equines translating in a dry cough. The infection generally abates within a few days. Mainly airborne transmission via aerosols. The virus rapidly spreads through susceptible horse populations. Fomites may play a role. (28) |  |
|  |  | *swine influenza virus*  *(SIV)* | swine influenza |  | In pigs, the infection of the upper respiratory tract associates with nasal secretions, paroxysmal coughing and sneezing. The virus concentration in nasal secretions is high and recovered pigs may continue to shed virus for several days. The infection lasts about six days. The virus can be shed for 30 days after infection and has been recovered from clinically normal animals. The virus transmits mainly through direct contact. Spread to new areas and farms by the movement of infected pigs or carrier people. The virus resists low temperatures; winter epizootics. (29, 30) |  |
| *Paramyxoviridae* | (-)ssRNA | *avian*  *paramyxovirus*  *type 1*  *(APMV-1)* | Newcastle disease |  | The virus is responsible for variable clinical signs in terrestrial poultry. Severe infections result in, respectively, respiratory, gastrointestinal, and nervous signs. Also clinically normal birds may carry the virus. Mainly aerogene and fecal-oral transmission. The virus survives well at ambient temperatures in feces. (31, 32) |  |
|  |  | *peste des petits*  *ruminants virus*  *(PPRV)* | peste des petits ruminants |  | The virus is genetically identical to the rinderpest virus. The clinical signs in sheep and goats include nasal discharge, erosion of buccal mucosa, diarrhea and pneumonia. Mucopurulent respiratory discharge may persist for up to fourteen days. No carrier state. Virus transmission is mainly via aerosol or through direct contact. Also fomites spread the infection. (33) |  |
|  |  | *Rinderpest virus*  *(RPV)* | Rinderpest |  | The virus is genetically identical to the peste des petits ruminants virus. In cattle and buffaloes the clinical signs comprise oculonasal discharge, lesions of the buccal mucosa, protracted salivation, and diarrhea. The virus is excreted mainly during the first week of clinical disease. There are no carriers. The virus transmits through direct, close contact. Fomites are not viable means of transmission. (34) |  |
| *Picornaviridae* | (+)ssRNA | *avian encephalitis virus*  *(AEV)* | avian encephalo-myelitis |  | The infection in young chicken and turkeys is characterized by central nervous system signs. The virus is neurotropic, enterotropic, plus establishes in the proximal genital tract. The virus is responsible for congenital infection in chicken. Infected chicken shed virus for several weeks. In adults carriers may result. The virus transmits both horizontally and vertically. Virus transmission via the egg occurs in breeder flocks. Hatching chicks may shed virus in feces and infect in-contact birds. The virus is environmentally resistant. (35) |  |
|  |  | *enterovirus*  *encephalomyelitis*  *(PEV1)* | porcine polio-encephalomyelitis |  | The virus is a gut inhabitant of pigs. The virus is excreted in feces and oral secretions. Clinically recovered animals may continue to shed virus for up to seven weeks. Fecal-oral cycle. The virus may survive in the environment for three months. (36) |  |
|  |  | *foot and mouth*  *disease virus*  *(FMDV)* | foot and mouth disease |  | Infections in mammals are characterized by vesicles on the foot, buccal mucosa, and, in female hosts, teats. During incubation, virus may be present in milk and semen. Upon the onset of clinical signs virus may be present in breath, saliva, feces, and urine. In cattle, the carrier stage may last up to six months. Transmission is via aerosols, through direct contact, or involves fomites. In pigs, swill feeding may trigger an outbreak. Transmission may also result from artificial insemination with contaminated semen. (37, 38) |  |
|  |  | *swine vesicular*  *disease virus*  *(SVDV)* | swine vesicular disease |  | The virus causes a highly contagious disease in pigs, indistinguishable from foot-and-mouth disease in pigs. The infection is characterized by the formation of vesicles on the feet, lower limbs, and snout. Virus excretion from nose and mouth usually stops within two weeks. Virus continues to be shed in feces for up to three months. The spread within the herd is rapid, mainly resulting from direct contact. Swill derived from infected pigs is often responsible for a primary outbreak. The virus is environmentally resistant and survives for 560 days in lymph nodes in ham. (39, 40) |  |
| *Poxviridae* | dsDNA | *avipoxvirus;*  *fowlpox virus*  *(FWPV)* | Fowlpox |  | In chicken and turkeys the virus causes dry cutaneous lesions or diphteritic, wet nodules in mouth and trachea. The course of the infection is protracted. The virus transmits during close contact or through skin abrasions. Biting insects may serve as mechanical vector. The virus slowly spreads in poultry flocks. The resistant virus is abundantly present in the skin lesions. (41) |  |
|  |  | *capripoxvirus;*  *sheep and goat*  *pox virus*  *(SGPV)* | sheep and goat pox |  | The virus causes an acute, contagious disease in sheep and goats characterized by oculonasal discharge and pox lesions, on the skin and on the respiratory and alimentary tract mucosae. The skin lesions take weeks to heal. Transmission is direct or indirect. The virus remains infective in scabs for two months or for six months in the environment. (42, 43) |  |
|  |  | *lumpy skin*  *disease virus*  *(LSDV)* | lumpy skin disease |  | The virus causes cutaneous lesions and crusts in cattle and buffaloes. Nodules develop over the entire body. Pox lesions may develop also in the mucosae of the respiratory and alimentary tract. The virus may be present in oculonasal discharges, milk and semen. Virus shedding may be prolonged. Pregnant cows may abort. The principal method of transmission is mechanical by biting insects. The virus survives for a month in skin lesions and for several months in dried scabs. (44) |  |
|  |  | *parapoxvirus;*  *contagious pustular*  *disease virus*  *(CPDV)* | contagious pustular dermatitis, contagious ecthyma, orf |  | The virus causes a highly contagious disease in sheep and goats. Skin lesions occur on the mouth, muzzle, udder, and feet. Most infections heal in three to six weeks. The virus may remain infective on wool and hide up to one month after recovery. (45) |  |
| *Reoviridae* | dsRNA | *bluetongue virus*  *(BTV)* | Bluetongue |  | In sheep, the primary livestock host, the mostly transient infection associates with ulceration of digestive and respiratory mucosae. The tongue may turn cyanotic. Virus may be encountered in semen. Also abortion and birth of malformed lambs occur. Arbovirus transmitted by *Culicoides* midges. Infected midges remain infective for life. Virus may be encountered in semen. No epithelial virus shedding. The virus is environmentally labile. (46, 47) |  |
| *Retroviridae* | (+)ssRNA-RT | *avian leukosis virus*  *(ALV)* | avian leukosis |  | In chicken the virus causes subclinical infection, leukemia-like, proliferative disease of the hemopoietic system, clonal malignancies of the bursal-dependent lymphoid system, or myeloid leukosis. An aggressive form of myeloid leucosis emerged mid 1990s in broiler breeders. The virus may be shed into albumen or yolk. Congenitally infected chicks remain viremic for life. Horizontal transmission may result from direct contact or indirectly by exposure to virus in the environment. Virus may be present feces, saliva, and desquamated skin; virus survival outside the host body is restricted to a few hours. (48) |  |
|  |  | *bovine leukemia virus*  *(BLV)* | enzootic bovine leukosis |  | The virus causes enzootic leukosis in cattle and buffaloes. The virus is responsible for lymphomas in two-five percent of infected animals, mostly three-five years old. Lymphomas are fatal. The main transmission is lactogenic. Transmission is also trans-placental, perinatal, iatrogenic, or involving bloodsucking insects. There are no epithelial transmission modes. (49, 50) |  |
|  |  | *caprine arthritis-*  *encephalitis virus*  *(CAEV)* | caprine arthritis / encephalitis |  | This lentivirus causes a contagious disease of small ruminants, mainly goats, and is identical to MVV. The virus mostly causes asymptomatic infections. Encephalomyelitis may occur in two-six months-old kids. Chronic polyarthritis is the main syndrome in adult goats. The disease is always progressive. The virus may also cause pneumonia or mastitis. The main transmission is via ingestion of colostrum or milk. The virus has been detected also in semen, respiratory secretions and feces. Fomites do not play a significant role as the virus is fairly labile. (51, 52) |  |
|  |  | *equine infectious*  *anemia virus*  *(EIAV)* | equine infectious anemia, swamp fever |  | This lentivirus causes a mostly subclinical infection in equines. Occasionally, progressive loss of conditions and anemia may be observed. The virus is transmitted by Tabanidae and other biting flies, mechanically. Iatrogenic, trans-placental, and lactogenic virus transmission is also possible. The virus survives less than four hours in the insect. There are no epithelial transmission modes. (53) |  |
|  |  | *Jaagsiekte;*  *sheep retrovirus*  *(JSRV)* | ovine pulmonary adenocarcinoma, jaagsiekte |  | The virus is responsible for a pulmonary adenocarcinoma. Once tumor formation becomes evident the disease outcome will be fatal, mostly in animals of 3-4 years old. Clinical signs include dyspnea, wet cough, loss of weight, and oculonasal discharge. The virus is shed in lung secretions and transmits via aerosol. Only a minority of JSRV infected animals develop clinical disease during their commercial lifespan. Lymphoid cells/macrophages are initially infected. The virus is also shed in colostrum or milk. (54, 55, 56) |  |
|  |  | *Maedi-Visna virus*  *(MVV)* | Maedi-Visna,  ovine progressive pneumonia |  | See also the identical CEAV. This lentivirus causes a contagious disease of small ruminants, mainly sheep. The virus mostly causes asymptomatic infections. Clinical signs may develop after two years of incubation and involve dyspnea and progressive emaciation or nervous forms of disease. The main transmission is via ingestion of colostrum or milk. The virus has been detected also in semen, respiratory secretions and feces. Fomites do not play a significant role as the virus is fairly labile. (52, 57) |  |
